# College of Dentists, England

**Published:** 1857-06

**Authors:** 


### COLLEGE OF DENTISTS, ENGLAND.

A new institution, having this title, has been recently organized in London. The general object is the advancement of dental scence, and to accomplish this end means wilbe provided for the professional education and examination of future practioners in the art. The mother country has been inexplicably slow in attending to this important matter, for such a step should have been made years ago. On this Continent the dental profession has not met with such unmerited indifference. Similar associations to the above have been long in operation in different cities of the United

States. And through their efficiency students are yearly sent out well versed in the departments of their art, and admirably qualified to perform the discharge of its various requirements with ability and success. The Anglican College has begun this Session a course of lectures upon Anatomy, Physiology, Therapeutics, and Microscopical Anatomy, Chemistry, &c. They intend shortly to procure a Charter of Incorporation from the Government, and thus become an established institution of the country. And we have no doubt, in time, their general usefulness will be fully recognized, and find an outlet through numerous channels intended to be ministers to the public weal. When will our Canadian fraternity band themselves together for a similar purpose? It is rather premature, probably, to expect that anything so extensive could yet be done; but we hope the time is not far distant when the increasing necessities of a rapidly multiplying population will require more practitioners than at present, and then the feasibility of a like incorporation will be entertained. In the meanwhile, we think, something in a less imposing way might be undertaken by the formation of a Dental Society, in which the members might, by mutual efforts, endeavor to elevate their professional position, and by joint contributions add to the common stock of knowledge, as well as carry out other matters of detail that would naturally suggest themselves on examination.—*Med. Chron.*

